# Feasibility and acceptability of advanced practice nursing in Lebanon: A convergent parallel mixed-methods study

**DOI:** 10.1016/j.ijnsa.2026.100570

**Published:** 2026-05-22

**Authors:** Joy Hanoun, Pascale Beloni, Jean Toniolo

**Affiliations:** a Inserm U1094, IRD UMR270, Univ. Limoges, CHU Limoges, EpiMaCT - Epidemiology of Chronic Diseases in Tropical Zone, Institute of Epidemiology and Global Health – Michel Dumas, OmegaHealth, Limoges, France; b University Department of Nursing Science, Faculty of Medicine, Univ. Limoges, Limoges, France; c Department of Nursing Sciences, Faculty of Public Health, Antonine University, Hadat–Baabda Campus, Hadat–Baabda, Lebanon; d University Hospital of Limoges, Limoges, France

**Keywords:** Advanced practice nursing, Nurse practitioner, Primary health care, Chronic disease management, Health policy, Lebanon

## Abstract

**Background:**

Lebanon is facing a severe health system and workforce crisis, characterized by nurse emigration, service fragmentation, and a rising burden of chronic diseases. Advanced practice nursing may help strengthen access, continuity, and quality of care; however, these roles are not formally established in Lebanon.

**Objective:**

To assess the feasibility and acceptability of introducing APN roles in Lebanon, identify perceived barriers and enabling conditions, and inform policy and implementation planning.

**Design:**

Convergent parallel mixed-methods study informed by the participatory, evidence-based, patient-focused process for advanced practice nursing framework.

**Setting(s):**

Hospitals and primary care centers in Lebanon.

**Participants:**

Health professionals, adult service users, and key stakeholders involved in or affected by APN implementation.

**Methods:**

Quantitative data were collected using structured questionnaires administered to health professionals and adult service users and analyzed descriptively using proportions with 95% confidence intervals (Cls) and exploratory subgroup comparisons. Qualitative data was generated through key informant interviews and focus groups with relevant stakeholders and analyzed thematically. Quantitative and qualitative findings were integrated during the interpretation stage using a side-by-side comparison of convergence, complementarity, and divergence.

**Results:**

Most health professionals supported the introduction of advanced practice nursing roles (87.7%; 95% CI 81.4–92.1), and almost all anticipated benefits for chronic disease management and care coordination (99.3%; 95% CI 96.2–99.9). Most service users reported a willingness to receive follow-up from an advanced practice nurse (97.1%; 95% CI 89.9–99.2), and 92.6% (95% CI 83.9–96.8) anticipated improved access in underserved areas. Qualitative findings reinforced perceived benefits for prevention, continuity of care, and equity, while identifying major implementation barriers, including the absence of a clear legal and regulatory framework, unresolved scope-of-practice and reimbursement issues, limited public and professional awareness, insufficient educational pathways, and anticipated resistance from some physicians.

**Conclusions:**

Stakeholders regarded advanced nursing practices in Lebanon as acceptable and potentially beneficial, particularly for chronic disease care and access in underserved areas. However, implementation may require regulatory clarification, investment in master-level education, interprofessional engagement, and context-sensitive policy planning.

**Social media abstract:**

Stakeholders in Lebanon support advanced practice nursing, but legal, educational, and system barriers still limit implementation.

**What is already known**

• Advanced practice nursing can improve access to care, care coordination, and selected patient and service outcomes in many health systems.
• Lebanon is facing major health-system pressures, including workforce shortages, nurse emigration, and a growing burden of chronic diseases.
• Advanced practice nursing roles are not formally established or operationally regulated in Lebanon.

Alt-text: Unlabelled box dummy alt text

**What this paper adds**

• In Lebanon, stakeholders generally view advanced practice nursing as acceptable and potentially beneficial, particularly for chronic disease management, prevention, and continuity of care.
• High perceived value coexists with low baseline awareness and concerns regarding legal recognition, scope of practice, liability, reimbursement, and professional boundaries.
• The implementation of advanced practice nursing in Lebanon may necessitate regulatory reform, master's-level educational pathways, interprofessional engagement, and a phased approach.

Alt-text: Unlabelled box dummy alt text

## 1 Introduction

By 2030, non-communicable diseases will account for over 70% of global mortality, placing unprecedented demands on health systems already strained by demographic transitions, rising care complexity, and persistent workforce shortages (World Health Organization, 2023). These challenges highlight the likely need to redesign care delivery and optimize the deployment of highly qualified health professionals. Advanced practice nursing (APN) has emerged internationally as a strategic workforce model to enhance access, improve quality, and strengthen system responsiveness in healthcare (Kilpatrick et al., 2024).

The International Council of Nurses defines advanced practice nurses as registered nurses (RNs) with postgraduate education, advanced clinical expertise, expanded decision-making, and the capacity to integrate leadership, consultation, evidence-based practice, interprofessional collaboration into their roles (ICN and Schober, 2020).

Evidence across health systems suggests that advanced practice nurses can deliver care comparable to physician-led models, particularly in primary care and chronic disease management (Chou et al., 2020; Hayter et al., 2021; Sikka et al., 2021; Swan et al., 2015, 2021). Patient satisfaction and service indicators (e.g., access, timeliness) are often improved, while clinical outcomes (e.g., blood pressure and glycemic control) are generally similar and sometimes modestly better. However, effects vary by setting, population, scope of practice and study design, and cost findings remain heterogeneous (Woo et al., 2020; Kilpatrick et al., 2024).

Perceptions of APN roles are not uniformly positive. International researchers report ambivalence or resistance, particularly when scope of practice, accountability, supervision, and reimbursement remain unclear. Additional concerns include role overlaps, professional boundaries, and implementation burden. (Torrens et al., 2020; Leoni-Scheiber et al., 2022; De Raeve et al., 2023). In Europe, variability in role definitions and regulation can undermine legitimacy and contribute to mixed stakeholder acceptance (De Raeve et al., 2023). Despite this evidence base, the implementation of APN remains uneven worldwide and continues to face resistance. These findings underscore the need for context-adapted role design, interprofessional governance, and clear regulatory positioning when considering APN development in different health systems (Schober, 2016; De Raeve et al., 2023).

In France, APN roles have been formalized through master’s programs and regulation; however, their operationalization remains inconsistent and sometimes ambiguous despite strong institutional endorsement (Toniolo et al., 2024a). Comparable challenges occur in low- and middle-income countries, where weak governance and resource constraints limit APN formalization, despite evidence of benefits for access, rural equity, and task-shifting (Christmals and Armstrong, 2019).

Similarly in Gabon, researchers conducting a mixed-method study found high professional and patient acceptability of APN roles but pointed to the lack of a legal framework and inadequate recognition of nursing competencies as critical barriers. Success factors included master's-level education, formal credentialing, and sustained interprofessional dialogue with medical associations (Toniolo et al., 2024b). These regional experiences suggest that successful APN integration in fragile systems may require context-sensitive strategies combining policy reform, educational investment, and stakeholder engagement.

Within the Middle East and North Africa region, APN development remains limited and heterogeneous. Israel has formalized nurse practitioner pathways through national regulation and licensure, with roles in fields such as geriatrics, palliative care, and diabetes. While countries such as Jordan and Saudi Arabia have made progress in expanding advanced nursing roles, particularly in chronic disease management and primary care, most countries, including Lebanon, remain at the exploratory stage (Al-Maaitah et al., 2022; El-Jardali et al., 2021).

## 2 Background

Lebanon is undergoing a rapid epidemiological transition, with non-communicable diseases accounting for over 80% of deaths, including nearly half from cardiovascular diseases. (WHO, 2023). This burden occurs in the context of systemic fragility, political instability, and economic collapse, undermining the delivery of effective, equitable, and sustainable health services (El-Jardali et al., 2021). Lebanon’s hospital-based and largely private health system has been further weakened by successive crises, including the 2019 financial collapse, COVID-19, the Beirut port explosion, and workforce emigration (Bou-Karroum et al., 2022).

With approximately 1.7–1.9 nurses and midwives per 1000 people (Akl et al., 2022), far below global benchmarks, the country faces severe workforce shortages, particularly in primary care and rural areas. Despite the growing need for decentralized and continuous care, nurses in Lebanon remain largely confined to hospital roles with limited autonomy in primary and community settings (Hanoun et al., 2025). Currently, no national framework exists to regulate, train, or define APN roles, and strategic decision-making frequently excludes nursing perspectives.

Although reform efforts have emerged, progress remains slow and fragmented. Recent legal reforms (Law No. 221/2021 and subsequent implementing decrees) introduced a “specialized nurse” category for master’s prepared nurses but did not establish a protected APN title or expanded scope of practice (e.g., diagnosis, prescribing, reimbursement). Consequently, Lebanon-specific evidence on APN feasibility and acceptability remains limited (Hanoun et al., 2025).

Overreliance on physicians and hospital services constrains access and efficiency, while advanced practice nurses can safely assume expanded responsibilities in chronic disease management, including in resource-constrained settings (Pulcini et al., 2022; Woo et al., 2020). Simultaneously, the demand for preventive, continuous, and person-centered care is increasing, reinforcing the relevance of APN roles in Lebanon (WHO, 2023).

However, major barriers persist including the absence of an operational APN regulatory framework (protected titles, scope, liability, reimbursement), generating uncertainty around role boundaries and accountability. Hierarchical structures, limited interprofessional collaboration, and low public awareness further constrain APN implementation (El-Jardali et al., 2021; Fealy et al., 2023).

Compared with countries with established APN roles, defined competencies, and dedicated master’s programs, Lebanon remains in an exploratory phase. Ongoing academic initiatives aim to adapt international frameworks, such as the participatory, evidence-based, patient-focused process for APN; however, empirical evidence on stakeholder perceptions and implementation conditions remains scarce.

Lebanon’s health system is fragmented, with dominant private provision in hospital and outpatient care. The Ministry of Public Health acts as system steward and insurer of last resort. Primary care is delivered through private clinics and a national network of centers, many run by non-governmental organizations and municipalities. Weak referral systems and limited gatekeeping reinforce hospital-centered care and fragmented chronic disease management (World Bank, 2024; World Health Organization, Regional Office for the Eastern Mediterranean, 2024).

The regulatory environment for nursing should be distinguished from APN-specific regulations. Nursing practice is governed through national decrees that define categories and licensure requirements. Recent reforms have updated these classifications; however, they do not constitute a dedicated APN regulatory framework with an explicitly expanded scope of practice. Accordingly, in this study, “regulatory gaps” refers specifically to the absence of APN-specific legal recognition and operational scope, rather than the absence of nursing regulations as such.

### 2.1 Aim of the study

We explored the feasibility of introducing APN in Lebanon, with a focus on chronic disease management. Specifically, we examined stakeholders’ perceptions of APN relevance and acceptability, anticipated role functions and boundaries, and perceived facilitators and barriers to implementation. In this manuscript, “stakeholders” refers to health professionals and system leaders involved in shaping, implementing, or collaborating with APN roles (e.g., nurses, physicians, managers, educators, students, and directors), as well as adult service users and community members as potential end-users.

## 3 Methods

### 3.1 Research design and theoretical framework

We employed a single-phase convergent parallel mixed methods design in which quantitative and qualitative data were collected during the same study period, analyzed separately, and integrated during interpretation. The two strands were assigned equal priority (QUAN + QUAL). The quantitative strand provided breadth across stakeholder groups (acceptability, role functions, implementation readiness), whereas the qualitative strand provided explanatory depth on perceived boundaries, enabling conditions, and contextual constraints (Creswell and Plano Clark, 2018). Supplementary Material S1 presents the study design schematic and an overview of participants across both quantitative and qualitative strands.

Integration occurred at three levels: design alignment, protocol harmonization, and interpretation. Both strands were aligned around shared aims and a common conceptual scaffold based on participatory, evidence-based, patient-focused process for APN was constructed, which informed the survey instruments and qualitative guides. The quantitative and qualitative strands were analyzed independently and then integrated at the interpretation stage using (i) a convergence coding matrix aligned with the study aims and Participatory, Evidence-based, Patient-focused Process for APN framework constructs (acceptability/appropriateness, anticipated role functions and boundaries, and implementation barriers/enablers), and (ii) a joint display juxtaposing key quantitative indicators with corresponding qualitative themes and illustrative quotations to identify convergence, complementarity, and divergence and to derive integrated meta-inferences (Table 5). Because survey data were anonymous and datasets not linkable, integration was conducted at the level of stakeholder category and construct.

Mixed-methods research is increasingly used in nursing and health systems research to address the multidimensional nature of professional role development (Östlund et al., 2011; Halcomb et al., 2020). Internationally, such designs have been applied to examine the barriers to and facilitators of APN implementation across diverse contexts (Torrens et al., 2020; Toniolo et al., 2024a, 2024b).

This study was guided by the Participatory, evidence-based, patient-focused process for APN framework, which provides a structured, stakeholder-engaged approach to APN role development, implementation, and evaluation. Although first published in 2004, the framework continues to be widely applied to APN role development and implementation planning, and has been extended through PEPPA-Plus to provide more explicit guidance for evaluating APN role contributions across the stages of introduction, implementation, and long-term sustainability (Bryant-Lukosius and DiCenso, 2004; Bryant-Lukosius et al., 2016).

The Participatory, Evidence-based, Patient-focused Process for APN framework was selected because Lebanon is in the pre-implementation stage, with no APN roles formally regulated or routinely deployed. We aimed to establish foundational evidence on feasibility and acceptability across multiple stakeholder groups to inform role definition and implementation planning. In this study, the Participatory, Evidence-based, Patient-focused Process for APN framework was operationalized primarily through its upstream components: identifying priority population needs and service gaps relevant to chronic disease care; engaging stakeholders across micro-, meso-, and macro-levels; and eliciting perceived role functions, boundaries, barriers, and enabling conditions to inform context-adapted role specifications and implementation prerequisites. Accordingly, the findings are intended to inform subsequent phases of APN role design and pilot implementation rather than evaluate an established APN service.

The quantitative and qualitative strands were conducted in parallel. A survey was administered anonymously to eligible health professionals and adult service users, whereas qualitative participants were recruited purposively to ensure representation across stakeholder groups and settings. Because the survey was anonymous and the qualitative dataset was not linkable at the participant level, overlap between strands could not be quantified. Accordingly, the survey sample sizes reflect only questionnaire completion and are reported separately from the qualitative sample.

### 3.2 Sample and setting

The study was conducted in Lebanon across multiple regions and institutions, including public, private, and university hospitals, as well as nursing schools. Sites were purposively selected to ensure variation in geography, sector, and organizational type. At least one institution was included in each major region of Lebanon.

Lebanon’s health system is predominantly private and hospital-oriented, with primary care delivered through a national network of centers coordinated by the Ministry of Public Health, many operated by non-state actors. This structure informed sampling and interpretation.

Participants included nurses, physicians, hospital directors, medical directors, nursing directors, nursing educators, nursing students, adult patients, and community members. Patients were included to capture end-user acceptability and expectations regarding a hypothetical APN role, as APN is not currently implemented in Lebanon.

### 3.3 Sampling strategy, eligibility criteria, and recruitment

The quantitative strand used a multistage, non-probability sampling strategy. Study sites were selected purposively to ensure geographic and sector representation, including public, private, and university hospitals, nursing schools, and limited primary care settings. Within the sites, facility-based convenience recruitment was used: eligible health professionals and adult service users were invited to participate voluntarily, without compensation, and completed an anonymous questionnaire. Accordingly, the quantitative sample sizes reflect the number of completed questionnaires rather than probability-based denominators Supplementary Material Appendix D.

The qualitative strand used purposive maximum-variation sampling. Key informant interviews targeted decision-makers involved in regulation, workforce planning, and service organization, while focus groups were conducted by participants category to elicit profession-specific perspectives and reduce hierarchical inhibition.

The eligibility criteria were defined a priori to ensure that respondents had sufficient role exposure to provide informed perceptions regarding APN feasibility and implementation conditions in Lebanon. Nurses were required to have at least 3 months of post-hire practice experience in participating in hospitals or in rural or semi-rural settings where nurses commonly assume broadened responsibilities. This threshold was chosen to ensure minimal familiarity with local workflows and interprofessional dynamics while preserving inclusivity in the context of workforce instability. Physicians were required to be licensed and currently practicing in Lebanon. Nursing educators had to be affiliated with an academic or clinical teaching institution. Nursing directors or lead nurses in governance roles required at least 3 years of experience in quality management and team coordination. Medical directors were required to have at least 5 years of leadership experience and registration with the Lebanese Order of Physicians. Hospital directors were required to have at least 5 years of hospital management experience and formal training in health administration. Students were eligible if enrolled in a nursing program to capture perceptions of the future workforce and educational pathway needs. Patients and service users were eligible if they were adults attending participating hospitals or primary care centers during the study period, able to provide informed consent, and able to complete the questionnaire in Arabic, English, or French. Chronic disease status and regular physician follow-up were collected through self-report to characterize the sample and contextualize responses.

Although primary care access is a central policy rationale for APN development, health professional survey respondents were predominantly hospital-based, reflecting Lebanon’s hospital-oriented service delivery and the study’s recruitment sites. In the quantitative sample, sample, almost three-quarters of participants reported working in private hospitals, compared to the smallest percentage in primary care centers (Table 1). Quantitative findings should therefore be interpreted primarily as reflecting hospital-sector perceptions, and primary care–specific inferences should be made cautiously.

**Table 1:** Sociodemographic and professional characteristics of health professional survey participants (*N* = 146).

Variable	Category	*n*	% of total
Sex	Female	122	83.6
	Male	24	16.4
Profession	Nurse manager	27	18.5
	Nursing director	7	4.8
	Student	22	15.1
	Educator/Trainer	12	8.2
	Nurse	67	45.9
	Physician	11	7.5
Practice setting	Private hospital	89	72
	Public hospital	3	2.4
	Primary care center	2	1.6
	Long-term care center	6	4.8
	University	11	8.8
	NGO/School	13	10.4
Care unit	Critical care	28	22.5
	Surgical care	13	10.4
	Medical care	43	34.6
	Outpatient/Community	18	14.5
	Management/Education Not reported (student)	22 22	17.7
Age (years)	N / Min–Max / Mean ± SD	146 / 20–88 / 39.55 ± 15.10	
Years of experience	N / Min–Max / Mean ± SD	(valid n = 124) / 0–66 / 20.92 ± 13.89	

Note. Note: Percentages are based on the total sample (*N* = 146), unless otherwise indicated. SD = standard deviation; NGO = non-governmental organization.

### 3.4 Data collection

#### 3.4.1 Quantitative phase

Quantitative data were collected between November 2024 and March 2025, with a brief interruption due to an armed conflict. A structured questionnaire was administered using KoboCollect, which is well suited to field settings with intermittent internet access. Two survey versions were developed: one for health professionals and one for adult service users/community respondents.

Instrument development followed a staged process. First, target constructs were identified in alignment with our theoretical framework and international guidance on APN, including perceived acceptability and appropriateness; anticipated role functions and boundaries; perceived impact on access, quality, continuity, and equity; and perceived implementation prerequisites. Second, the items were drafted and adapted from prior APN implementation studies and relevant competency frameworks (Schwingrouber et al., 2021; Toniolo et al., 2024a, 2024b). The brief APN knowledge section uses a small set of items derived from Hamric’s APN competency domains as an operational scaffold. These items were cross-checked against the International Council of Nurses’ Guidelines on APN to ensure their contemporary relevance and coverage (ICN, 2020). Third, the draft instrument underwent an interdisciplinary review by nursing and public health researchers to assess content relevance, neutrality of wording, and contextual appropriateness in a pre-implementation setting.

Pilot testing and face-validity assessment were conducted as distinct steps. The draft questionnaire was first piloted with two nurses to assess the feasibility of administration, including item flow, completion burden, and KoboCollect functionality, resulting in minor wording and formatting revisions. The questionnaire and standardized APN role description were then prepared in Arabic, English, and French. Each language version underwent linguistic and face-validity pretesting to ensure conceptual equivalence and comprehensibility in the Lebanese context. Pretesting focused on the clarity of instructions, interpretation of key terms, cultural acceptability, and the neutrality of the APN description. Feedback was documented and used to refine wording across versions.

The questionnaire included sociodemographic and professional characteristics; baseline awareness of APN; closed- and open-ended items assessing perceived relevance, acceptability, anticipated role functions, and perceived barriers and facilitators to APN implementation; items addressing anticipated contributions to chronic disease management, access, quality, and continuity; and a brief knowledge assessment of core APN competency domains. To facilitate the appraisal of measurement rigor, the full English versions of the health professional and service user questionnaires are provided in Supplementary Material Appendix A.

For this pre-implementation feasibility study, operational definitions were standardized to minimize conceptual ambiguity. An RN was defined as a licensed nurse practicing within the current Lebanese regulatory scope and customary care models. An advanced practice nurse is a postgraduate-prepared RN, in line with International Council of Nurses guidance, with advanced clinical assessment and decision-making competencies, alongside leadership, consultation, evidence-informed practice, and interprofessional collaboration functions. Because the APN is not yet a formally regulated role in Lebanon, the study materials explicitly framed APN as an advanced nursing role operating within a defined scope and governance model, rather than as a substitute for physicians. To avoid persuasive framing, the description emphasized role complementarity, referral/escalation for complexity, and the dependence of autonomy-linked functions on future regulation and collaborative arrangements.

#### 3.4.2 Qualitative phase

Qualitative data was collected between December 2024 and March 2025 through semi-structured interviews and focus groups. Two complementary qualitative modalities were used to optimize both depth and breadth in a pre-implementation feasibility context. Semi-structured interviews were prioritized for senior governance and decision-making roles, who were better positioned to discuss potentially sensitive issues such as scope governance, liability, reimbursement, and interprofessional negotiation in a confidential format. Focus groups were conducted with bedside stakeholder categories, including nurses, physicians, and patients/service users, to elicit shared norms, contested assumptions, and interactional dynamics relevant to APN implementation. Focus groups were conducted by stakeholder category to facilitate homogeneity of discussion and reduce hierarchical inhibition.

The qualitative sample included 33 unique participants: six key informants interviewed individually and 27 participants across five focus groups. The focus group compositions were as follows: nurses (three groups with 15 participants in total); physicians (one group with five participants); and patients/service users (one group with seven participants). Survey participation was not required for qualitative participation, and no individual-level linkages between the survey and qualitative data were undertaken.

Key informants were selected purposively based on their formal roles with decision-making influence over regulation, workforce planning, education, and service organization. All sessions were conducted face-to-face in private hospital meeting rooms, audio-recorded with consent, and transcribed. The interviews lasted approximately 45–60 min. Two trained female researchers, one nurse and one doctoral student in public health, experienced in qualitative methods, conducted the focus groups. No prior relationship existed with the participants, who were informed about the study aim before participating.

At the beginning of each interview or focus group, participants were first invited to describe their existing understanding of nursing roles and advanced practice. When needed, the interviewer then provided the same standardized APN role description used in the survey to ensure a shared minimum reference point while avoiding normative statements about expected benefits. The interview guide explicitly distinguished prior beliefs and experiences from views expressed after receiving the description, and probes were used to elicit perceived risks, role boundaries, and implementation conditions. The full English interview/focus group guide is provided in Supplementary Material Appendix B.

To protect confidentiality in small group settings and avoid indirect identification, quotations from nurse focus groups were attributed at the focus group level rather than to individual participants. Thus, N1–N3 referred to the three nurse focus groups rather than individual nurses. Interview quotations are attributed by role, and other focus group quotations use anonymized speaker codes, where appropriate.

Saturation was assessed iteratively, along with ongoing transcription and preliminary analysis. After each interview or focus group, the qualitative researchers conducted structured debrief and produced an analytic memo capturing emergent concepts, negative cases, and probe needs for subsequent sessions. Transcripts were coded in chronological order, and a running saturation log was maintained to document newly emerging codes, refinements to existing codes and theme boundaries, and whether additional data contributed substantively to new conceptual insights or only illustrative depth. Saturation was evaluated within stakeholder categories and across the entire dataset. This was achieved when consecutive sessions yielded no new codes, no material revision of the thematic structure, and sufficient elaboration of the main themes. Decisions were made through peer debriefing and documented in an audit trail.

### 3.5 Data analysis and rigor

#### 3.5.1 Quantitative analysis

Quantitative data were analyzed descriptively using IBM SPSS Statistics, version 22.0. Quantitative variables are presented as means and standard deviations or medians and interquartile ranges, depending on the distribution. Categorical variables are presented as frequencies and percentages. Group comparisons were performed using chi-square tests, with statistical significance set at *p* < .05. Missing data were minimal (<5%) and handled using complete-case analysis.

To assess whether the standardized APN description may have introduced systematic measurement bias, we conducted prespecified sensitivity analyses. Key outcomes, including support for APN introduction, willingness to be followed by an APN, and support for more autonomous functions, such as independent consultation and prescribing or renewal, were stratified by prior awareness of APN and survey language version. Where cell sizes allowed, exploration multivariate logistic regression models were fitted to adjust for profession or stakeholder group, years of experience, practice setting, and the above indicators. These analyses are reported in Supplementary Material Table S1 and should be interpreted cautiously, given the size of some strata.

#### 3.5.2 Qualitative analysis

Qualitative data were managed using NVivo 11 Plus and analyzed thematically following Tuckett (2005). Given the multi-stakeholder design, the analysis adopted a multi-perspective strategy informed by methodological work on triangulating multiple perspectives (Vogl et al., 2018, 2019) and APN research applying multi-perspective qualitative approaches across professional groups (Porat-Dahlerbruch et al., 2024).

Coding proceeded in two stages. First, two researchers independently open-coded an initial subset of transcripts from each stakeholder category and both data collection modes to develop a shared codebook, including labels, definitions, inclusion and exclusion criteria, and exemplars. The codebook was iteratively refined through constant comparison and peer debriefing and then applied to the full dataset. Discrepant coding was resolved through discussion, with adjudication by a third reviewer as needed, and decisions were documented in an audit trail.

Second, to avoid treating heterogeneous perspectives as a single analytical unit, within-stakeholder summaries were produced before conducting cross-stakeholder comparisons using matrix displays by stakeholder category and theme/subtheme. This allowed the identification of convergence, complementarity, and divergence. Divergent or minority positions, such as conditional acceptance of APN autonomy or concerns regarding role overlapping, liability, and governance, were retained explicitly as contrasting cases rather than collapsed into consensus. Stakeholder category and data collection mode were retained as analytic attributes to assess whether themes were stakeholder-specific or cross-cutting.

A linked-case multiple-perspective design was not used because participants were recruited to represent stakeholder categories relevant to system-level feasibility rather than interconnected relational units, such as patient–clinician dyads. Therefore, the analytic aim was to identify implementation conditions and boundary negotiations at the system level while maintaining convergent and divergent perspectives explicit.

Rigor was enhanced through triangulation across stakeholder groups, methods, and researchers; maintenance of audit trails and analytic memos; and transparent reporting consistent with COREQ.

Owing to the interruption caused by armed conflict, data collection occurred in two phases. Exploratory comparisons indicated no major differences in participant responses between the pre- and post-conflict periods, thus supporting the comparability of the dataset.

### 3.6 Mixed-methods data integration

After separating quantitative and qualitative analyses, findings were integrated at the interpretation stage using a convergence coding matrix aligned with the study aims and PEPPA-informed constructs: acceptability, anticipated role functions and boundaries, and implementation prerequisites. A joint display juxtaposed quantitative indicators with qualitative themes and illustrative quotations to assess convergence, complementarity, and divergence. When divergence occurred, quantitative subgroup patterns and qualitative transcripts were revisited to identify conditionality, stakeholder segmentation, or contextual explanations. Because survey responses were anonymous, integration was conducted by construct and stakeholder category rather than at the individual participant level.

### 3.7 Ethical considerations

Ethical approval was obtained from the relevant Institutional Review Board (reference no. 1820–2024). All participants provided written informed consent before participation. Confidentiality and anonymity were maintained throughout the study. All procedures were conducted in accordance with the Declaration of Helsinki.

## 4 Results

### 4.1 Health professionals

#### 4.1.1 Participant profile

A total of 146 health professionals participated in the survey. The sample was predominantly female, with substantial professional experience. Almost half of the respondents were nurses, followed by nurse managers, students, educators/trainers, nursing directors, and a small proportion of physicians. Most respondents worked in private hospitals, whereas only a few were employed in public hospitals or primary care centers. Detailed sample characteristics are presented in Table 1.

### 4.2 Perceptions of APN

Overall, health professionals expressed favorable perceptions of APN. Endorsement was strongest for patient follow-up, prevention, and care coordination, and more limited for autonomous activities such as prescribing and conducting independent consultations (Table 2).

**Table 2:** Endorsement of specific advanced practice nursing functions among health professionals, overall and by professional groups (n = 146).

APN activity	Overall, Yes, *n* (%)	Educator/Trainer (n = 12)	Nurse manager (n = 27)	Nursing director (n = 7)	Student (n = 22)	Nurse (n = 67)	Physician (n = 11)	Pearson χ² (df=5)	p
Patient follow-up	144 (98.6%)	100.0	96.3	100.0	95.5	100.0	100.0	4.078	*p = .538*
Training peers	102 (69.9%)	75.0	96.3	28.6	50.0	71.6	54.5	20.228	p = .001 ^* †^
Conducting consultations	50 (34.2%)	41.7	44.4	0.0	22.7	34.3	45.5	7.096	*p = .214 ^†^*
Doing research	111 (76.0%)	75.0	85.2	71.4	77.3	76.1	54.5	4.135	*p = .530 ^†^*
Coordinating care pathways	114 (78.1%)	83.3	77.8	85.7	68.2	76.1	100.0	4.932	*p = .424 ^†^*
Clinical assessment	108 (74.0%)	66.7	70.4	71.4	68.2	80.6	63.6	3.059	*p = .691 ^†^*
Diagnosing some conditions	87 (59.6%)	50.0	55.6	71.4	68.2	61.2	45.5	2.707	*p = .745 ^†^*
Prevention	127 (87.0%)	91.7	96.3	71.4	72.7	88.1	90.9	7.965	*p = .158 ^†^*
Prescribing	28 (19.2%)	25.0	33.3	14.3	9.1	17.9	9.1	6.097	*p = .297 ^†^*
Renewing prescriptions	40 (27.4%)	33.3	33.3	28.6	13.6	29.9	18.2	3.463	*p = .629 ^†^*

Note. Values in the profession columns are the percentages of respondents within each professional group who answered 'yes.' χ² = chi-square statistic. Pearson's chi-square test was used to assess differences across professional groups. * *p* < .05. ^†^ Results should be interpreted with caution due to small subgroup sizes in some professional categories.

A clear majority also supported the development of a master’s-level APN pathway and the formal recognition of advanced practices already performed informally by experienced nurses. However, participants also identified major implementation barriers, particularly physician resistance, the absence of a clear APN-specific regulatory framework, and limited awareness of the role.

### 4.3 Perceived contributions to chronic disease management

Participants strongly endorsed APN contributions to chronic disease-related care. Functions directly relevant to chronic disease management were widely supported, including patient follow-up, prevention, care pathway coordination, and clinical assessment. Support for diagnosing selected conditions was moderate. Physicians tended to report lower endorsement of clinical assessment and diagnosing selected conditions than nurses, although differences between professional groups were not statistically significant.

### 4.4 Acceptability of specific APN functions

Acceptability varied substantially according to the level of anticipated autonomy. The highest endorsement levels were observed for patient follow-up, prevention, care coordination, research, and clinical assessment. In contrast, support was considerably lower for conducting consultations, renewing prescriptions, and prescribing. The profession-specific endorsement rates are presented in Table 2. Among the tested differences, only support for training peers differed significantly across professional groups *(p* = .001).

### 4.5 Awareness gaps and implementation barriers

Most participants reported never having heard of APN before the survey, indicating limited baseline awareness of the role. Awareness appeared higher among educators and managers than among bedside nurses and physicians. Survey findings suggested that support for APN was conditional on clearer regulation, role definition, and professional recognition.

## 5 Patients/service users

### 5.1 Profile and health status

Sixty-eight participants completed the service user survey. Most were women, and the majority lived in Mount Lebanon, followed by Beirut, Bekaa, and North Lebanon. Occupations varied across multiple categories. More than a third reported being regularly followed by a physician, and approximately one-quarter reported having chronic illness (Table 3).

**Table 3:** The sociodemographic and health-related characteristics of the patient survey participants (n = 68).

Variable	Category	*n*	% (valid)
Sex			
	Female	55	80.9
	Male	13	19.1
	Total (valid)	68	100.0
Specify your profession			
	Other (specify)	1	1.5
	Office employee	19	27.9
	Teacher	7	10.3
	Student (non-health)	12	17.6
	Paramedical	3	4.4
	Retired	3	4.4
	Unemployed	9	13.2
	Self-employed	14	20.6
	Total (valid)	68	100.0
Were they regularly followed up by a physician?			
	No	39	57.4
	Yes	29	42.6
	Total (valid)	68	100.0
Do you have any chronic diseases (e.g., diabetes, hypertension, or cancer)?			
	No	51	75.0
	Yes	17	25.0
	Total (valid)	68	100.0
Education level			
	Doctorate	4	5.9
	Bachelor’s degree or equivalent	29	42.6
	Master’s degree or equivalent	23	33.8
	Secondary/High school	9	13.2
	Technical or vocational	3	4.4
	Total (valid)	68	100.0
Place of residence			
	Beirut	13	19.1
	Mount Lebanon	43	63.2
	North	5	7.4
	Bekaa	7	10.3
	Total (valid)	68	100.0

**Note.** n = number of participants; % (valid) = percentages based on valid responses (missing data excluded).

### 5.2 Perceptions of APN and perceived systemic impact

Baseline awareness of APN was absent among all respondents. After exposure to the standardized role description, participants indicated highly-anticipated acceptability, endorsing APN involvement in quality of care, continuity of follow-up, and time devoted to patients. Most agreed that nurses could serve as a central pillar in chronic disease management and already possess relevant competencies. Participants also perceived potential system-level benefits, particularly regarding physician shortages and access to care in underserved areas. Acceptability remained conditional on appropriate training, regulation, and professional oversight.

## 6 Qualitative findings

The thematic analysis generated five cross-cutting themes related to the introduction of APN in Lebanon: (1) structural and economic constraints, (2) legal framework and professional recognition, (3) education and professional development, (4) autonomy and recognition, and (5) implementation prerequisites. An overview of these themes, together with indicative subthemes and exemplar quotations, is presented in Table 4. Additional illustrative quotations by stakeholder groups are provided in Supplementary Material Table S1.

**Table 4:** Overview of the five overarching qualitative themes, indicative subthemes, and exemplar quotations.

Overarching theme	Indicative subthemes (examples)	Exemplar quotation
Structural & economic constraints	Economic crisis/financing; shortages and workforce migration; regional inequities	“The economic crisis we are living through.” (Nurse1)
Legal framework & professional recognition	Legislative gap; protected title/scope; liability and governance	“What we lack is a law recognizing advanced practice nurses.” (Nurse3)
Education & professional development	Variable initial clinical preparation; specialization; continuing professional development	“Specialization should be introduced at university.” (Nurse 2)
Autonomy & recognition	Theoretical vs actual autonomy; interprofessional boundaries; sociocultural acceptability	“At university, we’re taught we are autonomous, but in practice this isn’t applied.” (Nurse 3)
Implementing prerequisites.	Institutional/legal bottlenecks; professional resistance; implementation prerequisites	“The real problem stems from overall health policy: the law must be enforced.” (Nurse3)

### 6.1 Structural and economic constraints

Participants described a fragile health system marked by political instability, economic collapse, and workforce emigration. These structural pressures were seen as both drivers of the need for APN and barriers to its implementation. As one hospital director noted:

“Public hospitals are particularly affected funding is lacking, equipment is outdated, and nurses can’t project themselves professionally anymore.” (Nurse 6)

### 6.2 Legal framework and professional recognition

Across stakeholder groups, the absence of a clear APN-specific legal and regulatory framework was identified as a central barrier. Regulation was viewed as a prerequisite for legitimacy, role clarity, liability protection, and interprofessional acceptance. As one nurse stated:

“What we lack is a law recognizing advanced practice nurses.” (Nurse 5)

### 6.3 Education and professional development

Participants emphasized the need for structured master’s-level training and clearer specialization pathways to support safe and credible implementation of APN roles. One nurse highlighted:

“Specialization should be introduced at university.” (Nurse 8)

### 6.4 Autonomy and recognition

Autonomy was described as both an opportunity and a source of tension. While some participants viewed APN as strengthening nursing leadership, others raised concerns about blurred professional boundaries. A nurse explained:

“At university, we’re taught we are autonomous, but in practice this isn’t applied.” (Nurse 10)

### 6.5 Implementation prerequisites

Participants identified several practical conditions for implementation, including legal reform, institutional support, public awareness, and interprofessional engagement. As one participant noted:

“The real problem stems from overall health policy: the law must be enforced.”(Nurse 8)

Although these themes were identified across the dataset, their salience varied by stakeholder group. Nurses emphasized the lack of recognition of existing informal practices, physicians stressed the need for role clarity and governance, and patients/service users expressed general trust in nurses while highlighting the importance of training and standards for acceptability.

### 6.6 Integration of findings

The convergent mixed-methods design enabled an integrated interpretation of the feasibility and acceptability of APN in Lebanon. Quantitative findings indicated high endorsement of APN once the role had been described: 87.7% of health professionals supported the introduction of APN, and 97.1% of patients/service users reported willingness to be followed by an APN. At the same time, baseline familiarity was limited, with 84.9% of health professionals and all surveyed patients/service users reporting no prior awareness of APN.

This pattern was reinforced by both strands. Quantitative endorsement was strong for functions related to follow-up, prevention, care coordination, and chronic disease management, whereas support for more autonomous functions, such as consultations, prescribing, and prescription renewal, remained much lower. Qualitative findings complemented these results by showing that support for APN was conditional on clearer regulation, role delineation, educational preparation, and interprofessional agreement. Overall, the integrated findings suggest that APN is perceived as potentially valuable for improving quality, continuity, and access to care, particularly in chronic disease management and underserved areas; however, implementation remains constrained by regulatory uncertainty, system fragility, and professional boundary negotiations. The joint display is presented in Table 5.

**Table 5:** Joint display of convergence, complementarity, and divergence between quantitative and qualitative findings.

Quantitative finding	Qualitative finding (illustrative quote)	Integrated interpretation	Direction
98.5% believe advanced practice nurses improve quality of care	“It’s the nurse who really knows my history, not the doctor.” (Patient 3)	Strong convergence: Advanced practice nurses are widely perceived as enhancers of care quality and continuity.	Convergence
74% anticipate physician resistance	“Without clear legislation, conflict with doctors is inevitable.” (Physician 5)	Interprofessional tensions have consistently been identified as a major barrier.	Convergence
Only 34.2%, 19.2%, and 27.4% of health professionals supported independent consultations, prescribing, and prescription renewal, respectively.	“At university we learn autonomy, but in hospitals we must ask for approval for everything.” (Nurse2)	Complementary alignment: Both strands indicate that nursing autonomy is constrained in practice; low endorsement of autonomous APN functions reflects anticipated/experienced limits linked to governance, liability concerns, and entrenched hierarchies. on nursing autonomy.	Complementarity
84.9% of professionals and 100% of patients had never heard of APN	“We hear about APN abroad, but here no one explains what it means.” (Nurse manager)	Lack of awareness is systemic; however, once explained, support becomes overwhelming.	Convergence
92.6% of patients believe advanced practice nurses could address medical deserts	“In my village, no doctor is available. A trained nurse could really help.” (Patient 1)	Both strands highlight APN as a strategic response to workforce shortages and inequities.	Convergence
87.7% support creation of a Master’s degree in APN	“A clinical master’s program could give meaning to the profession again.” (Director of Care 1)	Clear alignment on the need for structured academic pathways to legitimize advanced roles	Convergence
98.6% of health professionals endorsed patient follow-up, and 87.0% endorsed prevention; 97.1% of patients anticipated more consistent follow-up after the standardized APN description.	“The nurse reminds me to take my medication and see the doctor.” (Patient 2)	Reinforces the consensus that advanced practice nurses should lead prevention and long-term patient follow-up.	Convergence
67.8% cited the absence of an operational APN regulatory framework as a major barrier.	“Without a law that recognizes the advanced practice nurse, autonomy is just words.” (Nurse 3)	Both professionals and leaders view regulations as critical prerequisites for APN implementation.	Convergence
99% reported high support for advanced practice nurses in chronic disease management, whereas only 46% of physicians were favorable.	“They could help lighten our load in chronic cases, but boundaries must be clear.” (Physician 3)	Quantitative endorsement is tempered by physicians’ ambivalence regarding role clarity.	Complementarity
98.5% of patients/service users supported APN integration to improve quality of care	“If she is well-trained and experienced, I am fine with it. But there must be standards.” (Patient 2)	Trust in nurses is conditional on their competence and regulation, mirroring their professional concerns.	Complementarity

## 7 Discussion

We have provided key insights into the opportunities and challenges of implementing APN roles in Lebanon, a context marked by economic collapse, workforce shortages, and a growing burden of non-communicable diseases. These findings align with international evidence highlighting the potential of APN roles to enhance access, improve quality, and promote equity in healthcare (WHO, 2023; Swan et al., 2021). However, Lebanon’s fragile health system introduces specific constraints, including the absence of APN-specific legal recognition and scope of practice, persistent interprofessional hierarchies, and cultural preferences for physician-led care.

Patients expressed high baseline trust in nurses and highly anticipated the acceptability of APN-led follow-up once the role was specified. Importantly, this pattern should not be interpreted as an automatic transfer of trust from RN to advanced practice nurses. Rather, it suggests that existing relational trust may facilitate acceptance when competencies and role boundaries are clearly communicated. In this study, acceptability items were presented after participants received a standardized APN description distinguishing APN from routine RN practice in terms of education and competencies, while also clarifying its current lack of regulation in Lebanon.

Consistent with this interpretation, we found that both qualitative and qualitative findings indicated that acceptability was conditional. While support was high for follow-up, prevention, and care coordination, endorsement was substantially lower for autonomy-linked functions, such as independent consultations and prescribing. This pattern suggests that acceptance depends on clear scope, accountability, and regulation.

### 7.1 Advanced practice nurses as a response to the non-communicable diseases burden

Health professionals strongly endorsed the potential contribution of APN to chronic disease management and care coordination. This aligns with Lebanon’s epidemiological context, where non-communicable diseases account for a large share of mortality and care remains hospital-centered and physician-driven (WHO, 2023; El-Jardali et al., 2021).

Participants viewed APN roles as a pragmatic response to workforce shortages and gaps in continuity of care, particularly in underserved areas. This aligns with global recommendations promoting task-sharing strategies to optimize service delivery in resource-constrained settings (WHO, 2020). Similar findings have been reported across diverse contexts, including Europe and sub-Saharan Africa, where APN roles contribute to improved access to and continuity of care (Pulcini et al., 2022; Woo et al., 2020; Kilpatrick et al., 2024; Toniolo et al., 2024b).

### 7.2 The paradox of strong support but limited awareness

A central finding of this study was the coexistence of a high perceived value of APN and very limited baseline awareness. Most health professionals and all patients and service users reported no prior knowledge of APN before the survey.

Because most participants were unfamiliar with APN, findings reflect perceptions formed after exposure to a standardized description rather than pre-existing attitudes. Targeted communication strategies may, therefore, be needed to support role understanding during implementation.

Although the description was designed to be neutral and provide a shared reference point, responses should be interpreted as reflecting acceptability under minimal standardized information. In this context, strong endorsement alongside concerns about autonomy and regulation is understandable. Stakeholders may support APN roles for continuity and workload redistribution while remaining cautious about expanded clinical authority.

Similar patterns have been observed in the early stages of APN development in Europe, where inconsistent regulation and limited public understanding have affected the legitimacy of the profession (Kroezen et al., 2018; De Raeve et al., 2023). Consistent with international guidance, formal legal recognition of APN, encompassing protected titles, defined scopes of practice, and reimbursement pathways, is essential for sustainable implementation (Schober, 2016).

### 7.3 Lessons from international experiences

In France, APN roles have been formalized through master's-level education and regulatory frameworks; however, implementation remains variable across regions and settings (Devictor et al., 2022; Schwingrouber et al., 2023). Similar patterns show that regulatory endorsement alone is insufficient without investment in education, interprofessional dialogue, and organizational readiness (Schober, 2016; De Raeve et al., 2023).

A phased implementation strategy may be appropriate in Lebanon, beginning with pilot programs targeting chronic disease management in controlled settings, such as university hospitals, followed by gradual expansion to primary care. Similar approaches have been effective in other health systems undergoing transitions (Maier et al., 2017; Toniolo et al., 2024a, 2024b).

### 7.4 Interprofessional tensions and cultural expectations

Interprofessional dynamics have emerged as a central implementation challenge. Anticipated physician resistance, blurred professional boundaries, and liability concerns suggest that APN implementation must be negotiated around scope and authority. The low endorsement of autonomy-linked functions reflects concerns regarding accountability, governance, and role delineation and is consistent with patterns reported in the Middle East and Europe during early APN adoption (El-Jardali et al., 2021; Fealy et al., 2023). Addressing these issues may require structured interprofessional dialogue, clear regulation, and phased role introduction to build trust.

Strategies to address these challenges include interprofessional education, development of clear clinical protocols, and regulatory clarification (Halcomb et al., 2023; Sikka et al., 2021). These approaches may help shift professional relationships from hierarchical to collaborative care models.

In contrast, patient participants expressed high levels of trust in nurses once the APN role was explained. However, this trust appeared conditional on training, standards, and oversight, and cultural preferences for physician-led care persisted in some contexts, particularly in rural areas. Public communication strategies may therefore be required to support broader societal acceptance (Al-Maaitah et al., 2022).

### 7.5 Contribution to global knowledge

We have contributed to the limited evidence on APN development in the Middle East by providing context-specific insights into regulatory, cultural, and interprofessional dynamics in a fragile health system.

From a conceptual perspective, the findings reflect the multilevel nature of APN implementation. Macro-level determinants include legal recognition, reimbursement mechanisms, and the scope of practice; meso‑level determinants include organizational governance, role clarity, and educational pathways; and micro-level determinants include interprofessional collaboration, boundary negotiation, and patient trust.

This multilevel pattern aligns with integration-oriented models of APN and supports the relevance of using the Participatory, Evidence-based, Patient-focused Process for APN framework for upstream role development in pre-implementation contexts, suggesting that integration frameworks may be useful for guiding future evaluations of APN implementation and sustainability (Porat-Dahlerbruch et al., 2022).

### 7.6 Study limitations

This study has several limitations that should be considered when interpreting the findings.

First, the use of self-report data may have introduced social desirability bias, particularly in a context marked by economic instability and heightened professional uncertainty. Although participation was voluntary and anonymous and questionnaires were multilingual, responses may still reflect socially desirable positions.

Second, the cross-sectional design captures perceptions at a single point in time and precludes causal inferences. This is particularly relevant in Lebanon’s rapidly evolving health system context, in which workforce dynamics, regulatory developments, and stakeholder perceptions may change over time.

Third, the study relied on a standardized, neutrally worded description of the APN role presented immediately prior to the perception items. While this approach was necessary, given the very low baseline awareness of APN, it may have introduced framing or priming effects. Thus, results reflect acceptability of a described role under minimal information rather than fully informed attitudes toward the complete APN scope.

Relatedly, limited prior awareness may have affected construct validity. Some participants may have interpreted the APN vignette as a general strengthening of nursing capacity rather than as a distinct, postgraduate-level clinical role with a defined scope, governance, and accountability. Although the inclusion of function-specific items (e.g., consultation, prescribing) partially mitigated this limitation by revealing lower support for autonomy-linked activities, high endorsement levels, particularly for broad system-level contributions, may have overestimated the acceptability of full-scope APN implementation in real-world settings.

Fourth, because the survey was anonymous and qualitative participation was independent, the datasets were not linkable at the participant level. As a result, integration relied on convergence across stakeholder groups and constructs rather than individual-level triangulation. Although consistent with the study design, this limits the ability to examine within-participant alignment between quantitative and qualitative responses.

Fifth, sectoral representation was imbalanced. Most healthcare participants in the quantitative sample were hospital-based, whereas primary care settings were underrepresented. Given that APN roles are often positioned within primary care and community settings, this may limit the transferability of the findings to those contexts.

Sixth, patient findings should be interpreted cautiously because APN is not currently implemented in Lebanon. Therefore, responses reflect anticipated acceptability based on a brief standardized description rather than experience with actual APN-delivered care. In addition, the proportion of participants reporting chronic diseases was relatively limited, which may constrain the applicability of the findings to populations most likely to benefit from APN roles.

Finally, rural and conflict-affected areas were underrepresented owing to security constraints during data collection. Consequently, the perspectives of populations facing the greatest access barriers may not be fully captured.

Despite these limitations, the use of a convergent mixed-methods design, including surveys, interviews, and focus groups across multiple stakeholder groups, strengthened the robustness of the findings. The convergence of the quantitative and qualitative results provided a nuanced and contextually grounded understanding of the feasibility and acceptability of APN in Lebanon.

## 8 Conclusion

Participants in Lebanon regarded APN roles as acceptable and potentially beneficial, particularly for chronic disease management and access in underserved areas. Implementation will likely require regulatory reform, master's-level educational pathways, interprofessional engagement, and context-sensitive policy planning. Importantly, patient endorsement reflected anticipated acceptability following standardized role information and should be interpreted considering low baseline awareness and potential framing effects.

APN role development in fragile health systems may be feasible when enabling conditions are deliberately and systematically established. Policy and implementation implications include (i) regulatory reform to define title protection, scope, accountability, and reimbursement pathways; (ii) investment in accredited master’s-level APN education aligned with international competency standards; and (iii) phased implementation supported by interprofessional governance and community-facing communication strategies. Researchers should consider evaluating pilot APN models using prospective designs and implementation outcomes (e.g., acceptability, appropriateness, feasibility, fidelity, and sustainability).

## Implications for nursing practice and policy

First, legislative reform may be required to provide advanced practice nurses with a clearly defined scope of practice, including prescriptive authority, liability coverage, and reimbursement. Without this framework (protected title, defined scope, liability, reimbursement), the role remains vulnerable to ambiguity and interprofessional conflict.

Second, academic institutions were identified by participants as central to APN development. Participants called for accredited master's-level programs aligned with international standards, emphasizing advanced clinical skills, leadership, and interprofessional collaboration. These educational pathways were viewed as essential to establishing the competence and legitimacy of APN roles in Lebanon.

Third, implementation should begin with pilot projects on chronic disease management, particularly within university hospitals and primary care centers. These initiatives would generate evidence on APN effectiveness in improving access, quality, and patient satisfaction, supporting scale-up.

Fourth, interprofessional collaboration was identified by participants as essential to mitigating resistance and clarifying professional boundaries. Participants called for clear protocols delineating APN responsibilities and joint training opportunities as strategies to support role integration across professional groups.

Finally, patient participants expressed strong willingness to receive care from advanced practice nurses after exposure to a standardized role description. Some of them noted reservations related to unfamiliarity with the role, which may reflect limited public awareness rather than cultural resistance. Targeted campaigns that highlight APN competencies, patient success stories, and benefits of nurse-led care may be able to strengthen societal support and accelerate its adoption.

In summary, advancing APN integration in Lebanon may require a combination of legal reform, educational investment, pilot implementation, interprofessional collaboration, and public engagement. These steps are mutually reinforcing and may need to be pursued in a coordinated manner to support the introduction of APN in Lebanon.

## Declaration of generative AI and AI-assisted technologies in the writing process

No generative AI or AI-assisted technologies were used in the preparation, writing, or editing of this manuscript.

## CRediT authorship contribution statement

**Joy Hanoun:** Writing – original draft, Visualization, Software, Project administration, Methodology, Investigation, Formal analysis, Data curation, Conceptualization. **Pascale Beloni:** Writing – review & editing, Validation, Supervision, Methodology. **Jean Toniolo:** Writing – review & editing, Validation, Supervision, Methodology, Data curation.

## Declaration of competing interest

The authors declare that they have no known competing financial or personal interests that could have appeared to influence the work reported in this article. There are no financial relationships, personal relationships, or organizational affiliations that might raise questions of bias in connection with this manuscript.

All authors have reviewed and approved the final version of the manuscript. We affirm that the article presents our genuine findings and that no conflict of interest exists in any aspect of the research or publication process.

## Appendix Supplementary materials

Image, application 1

Image, application 2

Image, application 3

Image, application 4

Image, application 5

Supplementary Material Image, application 6
