# Supplementary material for: Feasibility and acceptability of advanced practice nursing in Lebanon: A convergent parallel mixed-methods study
Source: Int J Nurs Stud Adv. 2026 May 22;11:100570. doi: 10.1016/j.ijnsa.2026.100570 (PMC13251711; doi:10.1016/j.ijnsa.2026.100570)
Supplement: Supplementary file 1 [file mmc1.pdf]

Étude exploratoire des enjeux liés à l’évolution du métier d’infirmier au Liban: préalables à l’implantation de la formation d’infirmière en pratique avancée .

Invitation to Participate in a Research Study

You are invited to participate in a research study titled **"Exploratory Study on the Challenges Related to the Evolution of the Nursing Profession in Lebanon: Prerequisites for the Implementation of Advanced Practice Nursing Education"**, conducted by Ms. Joy Hanoun, PhD candidate at the University of Limoges within the EpiMaCT research team. This study complies with the protocol approved by the ethics committee.

The approved method for approaching participants is online data collection. The objective of the study is to explore the challenges and facilitators related to the evolution of the nursing profession and to assess the acceptability and potential impact of this evolution in the context of Lebanese hospitals.

This message invites you to read the consent form and decide whether you wish to participate in the study.

Key points to note:

- Participation is entirely voluntary.
- Completing the questionnaire will take approximately 10 to 15 minutes.
- Only the data provided in the questionnaire will be collected and analyzed.
- The research team will not have access to your name or contact information.
- The survey results will be published in a scientific article, available in print and electronic format, and presented at conferences.
- Participants and institutions will not be identified in the publications.
- The study involves no risks beyond those encountered in daily life.
- Data will be stored securely, and only the principal investigator will have access to it.
- Inclusion criteria apply to nurses with more than three months of professional experience.
- The target sample size is approximately 400 participants.
- You may save a copy of the informed consent form for your records.
- There will be no financial compensation for participating in this study.

The study's results will help propose recommendations for implementing advanced practice nursing in Lebanon, aiming to improve access to care and the quality of nursing services. The associated risks are minimal. Collected data will remain confidential and anonymous, analyzed solely for this research, in compliance with applicable regulations.

◯ OK

CONSENT

I declare that I have read and understood the information presented in this document. I have had the opportunity to ask questions and have received satisfactory answers. I understand that my participation is voluntary and that I can withdraw at any time without providing any justification. I consent to participate in this study.

◯ Yes

◯ No

Please indicate the first three letters of your family name.

____

Please indicate the first three letters of your first name.

____

Gender

- ○ Male
- ○ Female

Age

In years

____

Profession

- ○ Physician
- ○ Nurse
- ○ Nurse manager
- ○ Student
- ○ Trainer
- ○ Nursing Director
- ○ Other

If other, please specify.

____

Years of experience in the profession.

In years.

____

If you are a nursing student, which year of study are you in?

____

Current workplace location.

- Public hospital
- Private hospital
- Primary care center
- School
- Home care
- University
- Other

If other, please specify.

____

If other, please specify.

- Emergency
- Surgery
- Medicine
- Oncology
- Pediatrics / Neonatology
- Obstetrics and Gynecology
- Psychiatry
- Anesthesia and Resuscitation
- Intensive Care
- Dialysis
- Other

If other, please specify.

____

Current state of the nursing profession in Lebanon.

Do you think nurses can be a cornerstone in the management of chronic diseases in Lebanon?

- Yes
- No

Do you think nurses in Lebanon currently have the skills to diagnose and manage chronic diseases?

- Yes
- No

In your opinion, could an advanced-skilled nurse address healthcare needs related to...

- ☐ Patient follow-up
- ☐ Peer training
- ☐ Conduct consultations
- ☐ Conduct research
- ☐ Coordinate care pathways
- ☐ Perform clinical assessments
- ☐ Diagnose certain conditions
- ☐ Conduct preventive care
- ☐ Prescribe
- ☐ Renew prescriptions
- ☐ Other

If other, please specify.

____

Knowledge of Advanced Practice Nurse (APN).

Have you heard about Advanced Practice Nurse (APN) in Lebanon?

- ○ Yes
- ○ No

If yes, can you specify its roles?

____

Do you think introducing advanced nursing practice in Lebanon would enhance the profession?

- ○ Yes
- ○ No
- ○ Not sure

In your opinion, could implementing advanced practice nurses help address the shortage of doctors in certain areas of Lebanon (rural and semi-rural areas)?

- ○ Yes
- ○ No

What advantages do you think an advanced practice nurse could bring? (check all that apply).

- ☐ Improved patient follow-up
- ☐ Increased time dedicated to patients
- ☐ Reduced workload for doctors
- ☐ Better prevention and diagnosis of chronic diseases
- ☐ Other

If other, please specify.

____

In what types of diseases could advanced-skilled nurses intervene effectively in Lebanon? (check all that apply).

Interventions for diabetes.

- ☐ Prevention
- ☐ Screening
- ☐ Diagnosis
- ☐ Initiation of treatments
- ☐ Monitoring

Interventions for breast cancer.

- ☐ Prevention
- ☐ Screening
- ☐ Diagnosis
- ☐ Initiation of treatments
- ☐ Monitoring

Interventions for breast cancer.

- ☐ Prevention
- ☐ Screening
- ☐ Diagnosis
- ☐ Initiation of treatments
- ☐ Monitoring

Interventions for other types of cancer.

- [ ] Prevention
- [ ] Screening
- [ ] Diagnosis
- [ ] Initiation of treatments
- [ ] Monitoring

Interventions for epilepsy.

- [ ] Prevention
- [ ] Screening
- [ ] Diagnosis
- [ ] Initiation of treatments
- [ ] Monitoring

Interventions for stroke.

- [ ] Prevention
- [ ] Screening
- [ ] Diagnosis
- [ ] Initiation of treatments
- [ ] Monitoring

Interventions for myocardial infarction.

- [ ] Prevention
- [ ] Screening
- [ ] Diagnosis
- [ ] Initiation of treatments
- [ ] Monitoring

Interventions for depression.

- [ ] Prevention
- [ ] Screening
- [ ] Diagnosis
- [ ] Initiation of treatments
- [ ] Monitoring

Interventions for hypertension.

- [ ] Prevention
- [ ] Screening
- [ ] Diagnosis
- [ ] Initiation of treatments
- [ ] Monitoring

Interventions for heart failure.

- [ ] Prevention
- [ ] Screening
- [ ] Diagnosis
- [ ] Initiation of treatments
- [ ] Monitoring

Interventions for kidney failure.

- [ ] Prevention
- [ ] Screening
- [ ] Diagnosis
- [ ] Initiation of treatments
- [ ] Monitoring

Interventions for dementia.

- [ ] Prevention
- [ ] Screening
- [ ] Diagnosis
- [ ] Initiation of treatments
- [ ] Monitoring

Interventions for COPD.

- [ ] Prevention
- [ ] Screening
- [ ] Diagnosis
- [ ] Initiation of treatments
- [ ] Monitoring

Interventions for asthma.

- [ ] Prevention
- [ ] Screening
- [ ] Diagnosis
- [ ] Initiation of treatments
- [ ] Monitoring

Interventions for cardiovascular diseases.

- [ ] Prevention
- [ ] Screening
- [ ] Diagnosis
- [ ] Initiation of treatments
- [ ] Monitoring

Interventions for Alzheimer's disease.

- [ ] Prevention
- [ ] Screening
- [ ] Diagnosis
- [ ] Initiation of treatments
- [ ] Monitoring

In which workplaces could advanced-skilled nurses be implemented?

- [ ] In a hospital center
- [ ] In a primary care center
- [ ] Educational and research institutions
- [ ] School health centers
- [ ] Public health organizations and NGOs
- [ ] Rehabilitation and long-term care facilities
- [ ] Home health services
- [ ] Other

If other, please specify.

____

Advanced nursing practices involve obtaining a master's degree. The International Council of Nurses (ICN) states that "a registered nurse practicing in advanced practice has acquired theoretical knowledge, decision-making expertise in complex situations, as well as the clinical skills necessary for the advanced practice of their profession." In practice, advanced nursing includes: - orientation, education, prevention, screening, or diagnosis activities - evaluation and clinical assessment procedures, technical acts, and clinical and paraclinical monitoring acts - prescribing health products not subject to medical prescription, ordering additional tests, renewing, or adapting medical prescriptions.

Do you think a master's degree in advanced nursing practice is a way to enhance the nursing profession?

- ○ Yes
- ○ No

Do you think a master's degree in advanced nursing practice opens new opportunities for the nursing profession?

- ○ Yes
- ○ No

Do you think a master's degree in advanced nursing practice promotes loyalty within the nursing profession?

- ○ Yes
- ○ No

Do you think a master's degree in advanced nursing practice provides legal recognition of existing practices?

- ○ Yes
- ○ No

Do you think a master's degree in advanced nursing practice could help address medical shortages in underserved areas?

- ○ Yes
- ○ No

Impact of Advanced Practice Nurses (APNs) on healthcare professionals and patients.

Do you think integrating advanced practice nurses would improve the quality of care in Lebanon?

- ☐ Yes
- ☐ No

Do you think advanced practice nurses could allow more time to be dedicated to patients?

- ○ Yes
- ○ No

Do you think advanced practice nurses could provide closer patient follow-up?

- ○ Yes
- ○ No

Do you think advanced practice nurses could provide more preventive care for patients?

- ◯ Yes
- ◯ No

How do you perceive the collaboration between doctors and advanced practice nurses?

- ☐ Positive, APNs could reduce doctors' workload
- ☐ Negative, it could create competency conflicts

Would working with an advanced practice nurse allow:

- ◯ To improve collaboration between medical and paramedical teams
- ◯ To provide a complementary perspective on care
- ◯ To increase the capacity for intervention when needed

Potential risks associated with the implementation of Advanced Practice Nurses (APNs).

What do you think are the main challenges or risks associated with the introduction of advanced practice nurses in Lebanon?

- ☐ Misunderstanding of the role of APNs
- ☐ Resistance from doctors
- ☐ Overlap of medical competencies
- ☐ Lack of a well-defined official status
- ☐ Underutilization of nursing skills
- ☐ Other

If other, please specify.

____

How could these challenges be overcome to successfully implement advanced practice nurses in Lebanon?

____

Additional comments: If you have suggestions or remarks regarding the introduction of advanced practice nurses in Lebanon, please share them here:

____
