# Supplementary material for: Feasibility and acceptability of advanced practice nursing in Lebanon: A convergent parallel mixed-methods study
Source: Int J Nurs Stud Adv. 2026 May 22;11:100570. doi: 10.1016/j.ijnsa.2026.100570 (PMC13251711; doi:10.1016/j.ijnsa.2026.100570)
Supplement: Supplementary file 2 [file mmc2.pdf]

Étude exploratoire des enjeux liés à l’évolution du métier d’infirmier au Liban: préalables à l’implantation de la formation d’infirmière en pratique avancée .

nvitation to participate in a research study
You are invited to participate in a study titled:

"Exploratory Study on the Challenges Related to the Evolution of the Nursing Profession in Lebanon: Prerequisites for the Implementation of Advanced Practice Nursing Training", conducted by Ms. Joy Hanoun, PhD candidate at the University of Limoges.

Objective:

To explore the challenges and facilitators related to the evolution of the nursing profession and to assess the acceptability and potential impact of this evolution in Lebanese hospitals.

Important Information:

Voluntary participation.
A questionnaire that takes 10 to 15 minutes.
Collected data will remain anonymous and confidential.
No additional risks beyond daily life.

Results will be published in scientific articles and presented at conferences.

Your participation will contribute to developing recommendations for implementing advanced practice nursing in Lebanon, aimed at improving access to care and the quality of nursing services.

○ OK

I declare that I have read and understood the information presented in this document. I have had the opportunity to ask questions and have received satisfactory answers. I understand that my participation is voluntary, and I can withdraw at any time without justification. I consent to participate in this study.

○ Yes

○ No

Gender

○ Male

○ Female

Age

In years

____

Specify your profession

- ○ Office employee
- ○ Teacher
- ○ Student
- ○ Retired
- ○ Self-employed
- ○ Unemployed
- ○ Other (please specify)

If other, please specify

____

Place of residence

____

Level of education

- ○ No diploma
- ○ Primary
- ○ Middle school
- ○ High school / Secondary school
- ○ Technical or vocational
- ○ Bachelor's degree or equivalent
- ○ Master's degree or equivalent
- ○ Doctorate

Do you suffer from a chronic illness (diabetes, hypertension, cancer, etc.)?

- ○ Yes
- ○ No

Are you regularly followed by a doctor?

- ○ Yes
- ○ No

Current state of the nursing profession in Lebanon

Do you think nurses can be a pillar in the management of chronic diseases in Lebanon?

- ○ Yes
- ○ No

Do you think nurses in Lebanon currently have the skills to manage chronic diseases?

- ◯ Yes
- ◯ No

In your opinion, could an advanced-skilled nurse address healthcare needs related to...

- ☐ Patient follow-up
- ☐ Peer training
- ☐ Conducting consultations
- ☐ Conducting research
- ☐ Coordinating care pathways
- ☐ Performing clinical evaluations
- ☐ Diagnosing certain conditions
- ☐ Carrying out prevention
- ☐ Prescribing
- ☐ Renewing prescriptions
- ☐ Other

If other, please specify

____

Knowledge of Advanced Practice Nursing (APN)

Have you heard of advanced practice nursing (APN) in Lebanon?

- ◯ Yes
- ◯ No

If yes, can you specify their roles?

____

Advanced nursing practices involve obtaining a master's degree. The International Council of Nurses (ICN) states that "a registered nurse practicing in advanced practice has acquired theoretical knowledge, decision-making expertise in complex situations, as well as the clinical skills necessary for the advanced practice of their profession." In practice, advanced nursing includes:

Orientation, education, prevention, screening, or diagnostic activities
Evaluation and clinical assessment procedures, technical acts, and clinical and paraclinical monitoring
Prescribing health products not subject to medical prescription, ordering additional tests, renewing, or adapting medical prescriptions

In your opinion, could implementing advanced practice nurses help address the shortage of doctors in certain areas of Lebanon (rural and semi-rural regions)?

- ○ Yes
- ○ No

What benefits do you think an advanced practice nurse could bring? (check all that apply)

- ☐ Improved patient follow-up
- ☐ Increased time dedicated to patients
- ☐ Reduced workload for doctors
- ☐ Better prevention and diagnosis of chronic diseases
- ☐ Other

If other, please specify

____

If you were ill, in what situations would you accept to be followed by an advanced practice nurse after an initial consultation with a doctor?

- ☐ Prevention
- ☐ Screening
- ☐ Monitoring the progression of the condition
- ☐ Initiation of treatments
- ☐ Monitoring

In which workplaces could advanced-skilled nurses be implemented?

- ☐ In a hospital center
- ☐ In a primary care center
- ☐ Educational and research institutions
- ☐ School health centers
- ☐ Public health organizations and NGOs
- ☐ Rehabilitation and long-term care facilities
- ☐ Home healthcare services
- ☐ Other

If other, please specify

____

Do you think an advanced practice nurse could help address medical shortages in underserved areas?

- ○ Yes
- ○ No

Impact of advanced practice nurses on healthcare professionals and patients

Do you think integrating advanced practice nurses would improve the quality of care in Lebanon?

- ☐ Yes
- ☐ No

Do you think advanced practice nurses could allow more time to be dedicated to patients?

- ○ Yes
- ○ No

Do you think advanced practice nurses could provide closer follow-up for patients?

- ○ Yes
- ○ No

Do you think advanced practice nurses could enable more preventive care for patients?

- ○ Yes
- ○ No

Additional comments: If you have suggestions or remarks regarding the introduction of advanced practice nurses in Lebanon, please share them here:

____
