# Supplementary material for: Feasibility and acceptability of advanced practice nursing in Lebanon: A convergent parallel mixed-methods study
Source: Int J Nurs Stud Adv. 2026 May 22;11:100570. doi: 10.1016/j.ijnsa.2026.100570 (PMC13251711; doi:10.1016/j.ijnsa.2026.100570)
Supplement: Supplementary file 3 [file mmc3.docx]

***Appendix B.*** *English semi-structured interview and focus group guide*

1. **In your opinion, what are the main limitations of the Lebanese healthcare system currently?**
   - Consider aspects such as infrastructure, availability of human resources (physicians, nurses), and economic and political conditions.
2. **How do you perceive the potential evolution of nursing practice in Lebanon?**
   - What recent or future changes could improve the nursing profession?
3. **In your current environment, do you think the nursing profession should evolve? If yes, to what extent in terms of competencies?**
   - Consider the development of nursing roles in primary care, chronic disease management, or hospital settings.
4. **In the Lebanese context, what role could nurses play in addressing medical deserts, particularly in rural or semi-rural areas?**
   - How could advanced practice nurses intervene in these regions?
5. **What major health challenges in Lebanon could the nursing profession help address?**
   - For example, chronic disease management (diabetes, hypertension), prevention, or patient education.
6. **In your opinion, what are the key factors to ensure that the evolution of the nursing profession in Lebanon is beneficial in the long term?**
   - Reflect on education, interprofessional collaboration, and regulation.
7. **What barriers or challenges do you foresee in the evolution of the nursing profession in Lebanon?**
   - Consider professional recognition, conflicts with other healthcare professions, or institutional limitations.
8. **Do you think the advanced practice nursing (APN) model could be effective in Lebanon? What would be the advantages or challenges of its implementation?**
   - Reflect on APN education and its potential impact on the Lebanese healthcare system.
9. **What interprofessional collaborations (between physicians, nurses, and other healthcare professionals) would be necessary to maximize the effectiveness of nurses in the Lebanese healthcare system?**
10. **How do economic and political crises affect the development of the nursing profession in Lebanon?**

- Consider the impact of healthcare workforce migration, medication shortages, and hospital funding challenges.

1. **To what extent are nurses in Lebanon recognized as key actors in primary healthcare management?**

- Consider public perception and the views of other healthcare professionals regarding nurses’ roles in patient follow-up and chronic disease management.
