# Supplementary material for: Feasibility and acceptability of advanced practice nursing in Lebanon: A convergent parallel mixed-methods study
Source: Int J Nurs Stud Adv. 2026 May 22;11:100570. doi: 10.1016/j.ijnsa.2026.100570 (PMC13251711; doi:10.1016/j.ijnsa.2026.100570)
Supplement: Supplementary file 4 [file mmc4.docx]

***Appendix C.****. Standardized advanced practice nurse role description*

**Definition of Advanced Practice Nursing (APN) – International Council of Nurses (ICN)**

According to the International Council of Nurses (ICN), an Advanced Practice Nurse (APN) is a registered nurse who has acquired expert knowledge, complex decision-making skills, and advanced clinical competencies through specialized education and training. These competencies enable APNs to provide high-quality care across a range of clinical settings.

APNs practice in an expanded and autonomous role, which often encompasses responsibilities in clinical care, research, education, consultation, and leadership.

**Key Characteristics:**

1. **Advanced Education:**
   APNs hold an academic qualification at the master’s level or higher in nursing or a relevant clinical specialty.
2. **Autonomous Practice:**
   APNs can practice independently, including diagnosing, treating, prescribing, and managing patient care, while maintaining collaborative relationships with other healthcare professionals.
3. **Expanded Decision-Making:**
   Their role involves advanced clinical assessment, implementation of complex interventions, and comprehensive care planning.
4. **Leadership and Mentorship:**
   APNs contribute to health policy development, support professional mentoring, and play a key role in improving standards of care.
5. **Global Role:**
   APNs act as agents of change and advocate for equitable access to healthcare within health systems worldwide.

**Reference:**
International Council of Nurses (ICN). *Guidelines on Advanced Practice Nursing*. 2020. Available at: <https://www.icn.ch>
