# Supplementary material for: Feasibility and acceptability of advanced practice nursing in Lebanon: A convergent parallel mixed-methods study
Source: Int J Nurs Stud Adv. 2026 May 22;11:100570. doi: 10.1016/j.ijnsa.2026.100570 (PMC13251711; doi:10.1016/j.ijnsa.2026.100570)
Supplement: Supplementary file 5 [file mmc5.docx]

**Supplementary Material Appendix D:** Overview of study participants across quantitative and qualitative strands .


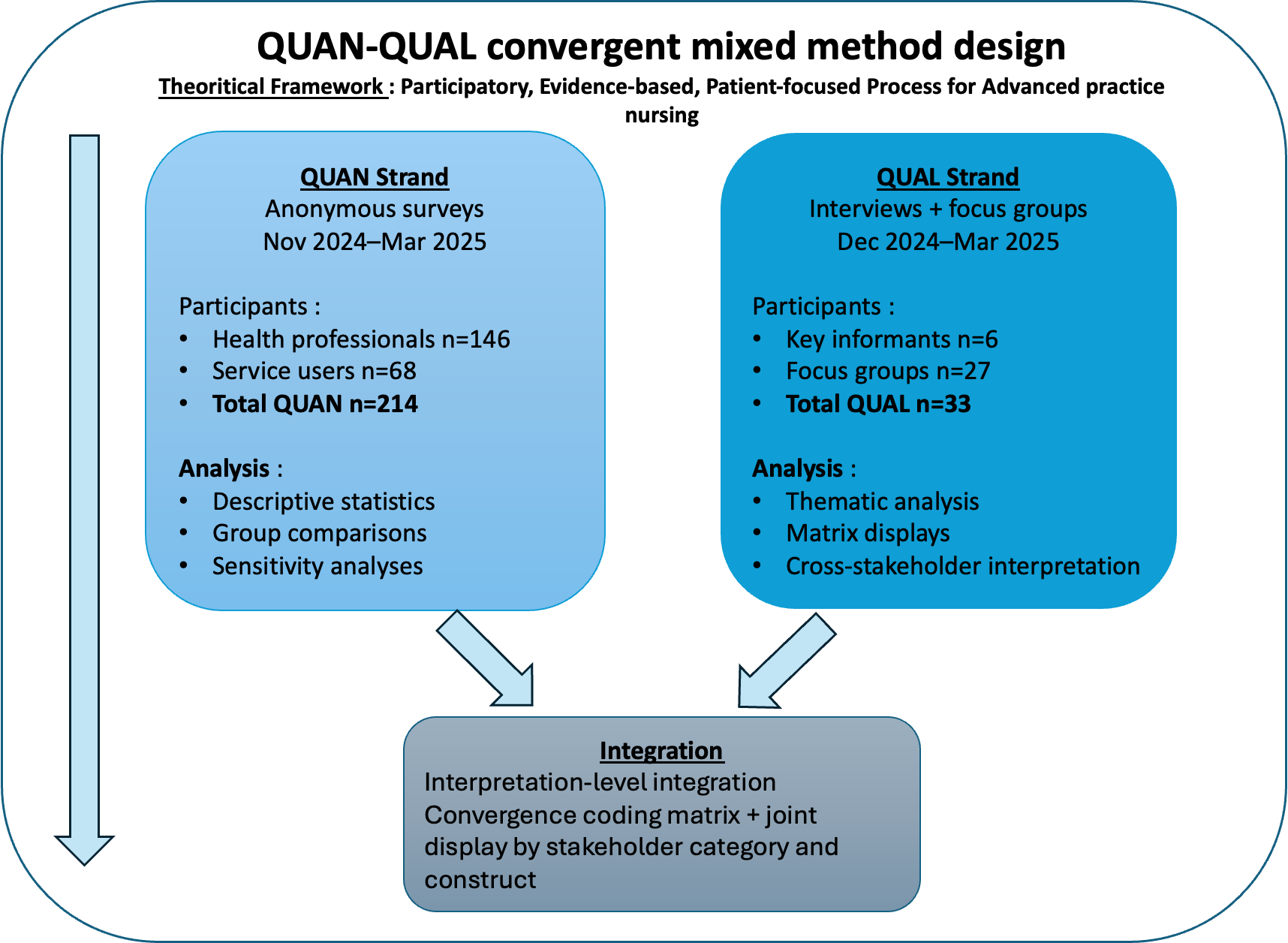


| **Strand** | **Component** | **Participant group** | ***n*** |
| --- | --- | --- | --- |
| **Quantitative** | Health professional survey | Nurses | 67 |
|  |  | Nurse managers | 27 |
|  |  | Students | 22 |
|  |  | Educators/Trainers | 12 |
|  |  | Nursing directors | 7 |
|  |  | Physicians | 11 |
|  |  | **Subtotal** | **146** |
|  | Service participant survey | Adult patients/service users | 68 |
|  | | **Subtotal** | **68** |
| **Qualitative** | Key informant interviews | Senior decision-makers | 6 |
|  | Focus groups | Nurses (3 groups) | 15 |
|  |  | Physicians (1 group) | 5 |
|  |  | Patients/service users (1 group) | 7 |
|  |  | **Subtotal** | **33** |
| **Total** |  |  | **247** |

**Note.** Qualitative participants were recruited independently from survey respondents. No individual-level linkage between strands was undertaken as surveys were anonymous.
