# Supplementary material for: Feasibility and acceptability of advanced practice nursing in Lebanon: A convergent parallel mixed-methods study
Source: Int J Nurs Stud Adv. 2026 May 22;11:100570. doi: 10.1016/j.ijnsa.2026.100570 (PMC13251711; doi:10.1016/j.ijnsa.2026.100570)
Supplement: Supplementary file 6 — Supplementary Material [file mmc6.docx]

| **Supplementary Table S1. Full stakeholder-by-subtheme qualitative quotation matrix (verbatim excerpts).** | | | |
| --- | --- | --- | --- |
| **Group** | **Main theme** | **Subtheme** | **Illustrative quote** |
| Nurses (Focus Group) | Structural & economic constraints | Economic crisis & financial impact | “Previously, there were many shortages […] even suppliers were affected. (N2)” |
|  |  | Economic crisis & financial impact | “The economic crisis we are living through. (N1)” |
|  |  | Health workforce migration | “There is a shortage on both the nursing and physician sides. (N3)” |
|  |  | Health workforce migration | “Many health professionals have emigrated. (N2)” |
|  | Legal framework & professional recognition | Legislative gap (APN not recognized) | “There is no official regulation specifying the nurse’s role. (N2)” |
|  |  | Legislative gap (APN not recognized) | “What we lack is a law recognizing advanced practice nurses. (N3)” |
|  | Education & professional development | Inadequate initial training | “Skill levels have regressed a lot across the board. (N1)” |
|  |  | Inadequate initial training | “Students do not have real hands‑on experience. (N1)” |
|  |  | Need for specialization / advanced practice | “Specialization should be introduced at university. (N2)” |
|  |  | Need for specialization / advanced practice | “Specialization could help stop the migration of experienced nurses. (N3)” |
|  |  | Limited access to continuing education | “We don’t even have time to attend trainings. (N1)” |
|  |  | Limited access to continuing education | “After the crisis, accessing training became impossible. (N3)” |
|  | Autonomy & recognition | Theoretical autonomy vs. real practice | “At university, we’re taught we are autonomous, but in practice this isn’t applied. (N3)” |
|  |  | Interdisciplinary collaboration (limited recognition) | “The nurse coordinates the entire paramedical team. (N2)” |
|  |  | Interdisciplinary collaboration (limited recognition) | “We work with other professionals, but our role is not respected. (N1)” |
|  |  | Sociocultural acceptability | “Patients do not always accept advice from a nurse. (N1)” |
|  |  | Sociocultural acceptability | “The physician must approve for the nurse to make decisions. (N2)” |
|  | Implementing advanced practice nursing (APN) | Legal & institutional bottlenecks | “The real problem stems from overall health policy: the law must be enforced. (N3)” |
|  |  | Professional resistance | “There is resistance from the medical profession. (N2)” |
|  |  | Professional resistance | “Physicians may slow down the evolution. (N3)” |
| Nursing Director & Hospital Director | Structural & economic constraints | Economic crisis / financing | “The main problem is economic, especially in public hospitals. (Public hospital director)” |
|  |  | Access to care | “A large share of the population lacks access to care. (Nursing director N2)” |
|  |  | Shortages & HR exodus | “The exodus of doctors and nurses abroad. (Public hospital director)” |
|  |  | Shortages & HR exodus | “Many nurses left after 2020. (Nursing director N2)” |
|  |  | Infrastructure disparities | “Some regions are completely underserved, like Akkar. (Nursing director N3)” |
|  | Education & professional development | Insufficient initial training | “Nurses trained during the crisis years mostly had online training. (Public hospital director)” |
|  |  | Heterogeneous initial training | “Not all universities emphasize clinical placements. (Nursing director N3)” |
|  |  | Continuing & specialized training | “Continuing professional development is essential. (Nursing director N1)” |
|  |  | Continuing & specialized training | “We need specialized training in oncology and wound care. (Nursing director N2)” |
|  | Strategic nursing role | Address medical deserts | “A professional nurse can deliver primary care. (Public hospital director)” |
|  |  | Innovative community care | “Nurses can provide community care via medical buses. (Nursing director N3)” |
|  | Empowerment & APN | Autonomy in primary care / protocols | “Nurses should manage primary care and adjust treatments under protocols. (Nursing director N2)” |
|  |  | Structuring the profession | “Advanced practice will help structure the profession. (Nursing director N1)” |
|  | Barriers | *Lack of legislative framework* | “The current legislative framework does not mandate professional autonomy. (Nursing director N)” |
|  |  | *Lack of legislative framework* | “Advanced practice needs clearer legal definition. (Nursing director N2)” |
|  |  | Résistances internes / interprofessionnelles | “There is resistance from physicians and sometimes even management. (Nursing director N3)” |
|  |  | Professionalism & ethics | “Ethical standards have declined; many only care about getting paid. (Public hospital director)” |
|  | Keys to APN development | Institutional recognition & leadership | “The Order must be strong enough to enforce advanced practice. (Nursing director N2)” |
|  |  | Leadership | “We need strong nursing leadership to negotiate with management. (Nursing director N3)” |
|  |  | Technology & innovation | “AI and technologies must be integrated into nursing care. (Nursing director N2)” |
|  |  | Technology & innovation | “AI is a companion to improve care. (Private hospital Director)” |
| Physicians (Focus Group) | Role and importance of nurses | Close to patients | “Nurses are closer to the patient than we are. (D1)” |
|  |  | Complementary support | “They provide support that physicians cannot always offer. (D3)” |
|  | Collaboration | Indispensable partnership | “We are indispensable partners. (D5)” |
|  |  | Teamwork is essential | “A physician cannot work without the team, especially the nurses. (D3)” |
|  | Training & competencies | Skills viewed as good but variable | “Most nurses are very well trained, but turnover is a problem. (D5)” |
|  |  | Experience reinforces training | “Their training is solid when reinforced by experience. (D4)” |
|  | Professional development | Need for continuing education | “The more you learn, the more you discover you know nothing. (D3)” |
|  | Workload & staffing | Staffing shortages affecting care quality | “Nursing staffing is insufficient, and this affects quality of care. (D5)” |
|  | Task delegation | *Tasks nurses can perform (within scope)* | “Examine ears, auscultate lungs, manage chronic wounds. (D3)” |
|  |  | *Tasks nurses can perform (within scope)*  *Conditional delegation with safeguards*  Feasibility of Advanced Practic… | “Insert a urinary or nasogastric catheter, perform emergency intubation. (D1)” |
|  |  |  | “Agrees with delegation provided there is good training. (D4)” |
|  |  | Need for legal protection / liability framework | “Yes, but a clear legal framework is needed to protect nurses. (D4)” |
|  | Advanced practice Nurse | Concept acceptability | “I would really like to see APNs develop. (D1)” |
|  |  | Acceptability under supervision | “The concept is well received if supervised by the care team. (D2)” |
|  |  | Priority areas | “Chronic disease management, preventive care, stable conditions. (D3)” |
|  |  | Priority areas | “Wound care follow‑up, pressure ulcers, diabetic feet, home care. (D5)” |
|  | Conditions for evolution | Specialized training (1–2 years) | “Add one or two years of study for nurses. (D2)” |
|  |  | *Clarify roles and responsibilities* | “Define boundaries and provide better legal oversight. (D4)” |
|  |  | *Strengthen interprofessional communication* | “Honest, regular communication is essential. (D3)” |
| Patients | Experience with nurses | Central role in care | “It’s the nurse who has followed me for years; she even manages my lab work. (P3)” |
|  |  | Central role in care | “In hospital, it’s always the nurse who takes care of me. (P5)” |
|  | Relationship & support | Trust & presence | “She supports us emotionally; she is always present. (P5)” |
|  |  | Continuous monitoring | “The nurse is the vigilant eye on the patient. (P6)” |
|  | Expressed needs | Understaffing & overload | “They work 12‑hour shifts; they are exhausted. (P7)” |
|  |  | Understaffing & overload | “We need many more nurses. (P3)” |
|  | Working conditions | Improve conditions / shifts | “The best conditions are at AUBMC, with 8‑hour shifts. (P2)” |
|  |  | Pay & engagement | “Low pay undermines their engagement. (P2)” |
|  | Acceptance of expanded role | Delegation of medical acts | “Of course, I consult my nurse before going to the doctor. (P3)” |
|  |  | Conditional trust | “If she has enough experience, I trust her. (P5)” |
|  | Conditions for APN | Experience & training | “She must have extensive experience and good training. (P2)” |
|  |  | Legal oversight | “Clear regulation is needed to avoid abuses. (P2)” |
|  | Cultural factors | Culture & education level | “In villages, patients may refuse to be followed by a nurse. (P1)” |
|  | Cultural factors | Culture & education level | “In more educated regions, it will be better accepted. (P2)” |
|  | Sociocultural change | Gradual change | “It takes time for people to accept nurses taking on a greater role. (P5)” |
|  | Proposed improvements | Selection & orientation | “Before entering nursing, personality testing should be done. (P7)” |
|  | Proposed improvements | Differentiation by training | “We should differentiate between those with a technical diploma and those with a master’s. (P3)” |
